# *Neisseria meningitidis* with decreased susceptibility to penicillin G and molecular characterization of the penA gene in strains isolated at University Hospital Centers of Casablanca and Marrakech (Morocco)

**DOI:** 10.11604/pamj.2024.47.56.42328

**Published:** 2024-02-08

**Authors:** Khadija Ait Mouss, Néhémie Nzoyikorera, Aziza Razki, Bahija Zaki, Nabila Soraa, Khalid Zerouali

**Affiliations:** 1 Department of Microbiology, Laboratory of Clinical Immunology, Inflammation and Allergy, Faculty of Medicine and Pharmacy, Hassan II University of Casablanca, Casablanca, Morocco,; 2 Bacteriology-Virology and Hospital Hygiene Laboratory, University Hospital Centre Ibn Rochd, 1 Rue des Hôpitaux, 20100, Casablanca, Morocco,; 3 Institut Pasteur du Maroc, 1 Place Louis Pasteur, 20360, Casablanca, Morocco,; 4 Faculty of Medicine and Pharmacy of Marrakech Cadi Ayyad University, Microbiology Department, Mohamed VI University Hospital Center, Marrakech, Morocco

**Keywords:** *Neisseria meningitidis*, CMI Peni G, Sanger sequencing

## Abstract

**Introduction:**

the laboratory diagnosis of meningococcal meningitis relies on conventional techniques. This study aims to evaluate the correlation between the reduced sensitivity to penicillin G of Neisseria meningitidis (N.m) strains and the expression of the altered PBP 2 gene.

**Methods:**

out of 190 strains of N.m isolated between 2010 and 2021 at the bacteriology laboratories of Ibn Rochd University Hospital Centre (IR-UHC) in Casablanca and the UHC Mohammed VI in Marrakech, 23 isolates were part of our study. We first determined their state of sensitivity to penicillin G by E-Test strips and searched for the expression of the penA gene by PCR followed by Sanger sequencing.

**Results:**

of all the confirmed cases of N.m, 93.15% (n=177) are of serogroup B, 75.2% (n = 143) are sensitive to penicillin G and 24.73% (n = 47) are of intermediate sensitivity. No resistance to penicillin G was observed. Reduced sensitivity to penicillin G in N.m is characterized by mutations namely F504 L, A510 V, I515 V, G541 N and I566 V located in the C-terminal region of the penA gene encoding the penicillin-binding protein 2 (PBP2) (mosaic gene).

**Conclusion:**

our study presents useful data for the phenotypic and genotypic monitoring of resistance to penicillin G in N.m and can contribute to the analysis of genetic exchanges between different Neisseria species.

## Introduction

*Neisseria meningitidis* (*N.m*) commonly known as meningococcus is a strictly human pathogenic bacterium; it is presented in the form of Gram-negative diplococci. It colonizes asymptomatically the nasopharynx and can be the cause of sepsis and/or meningitis. Invasive Meningococcal Infections (IMI) require urgent diagnosis, prompt and adequate antibiotic therapy. For more than forty years [1], penicillin has been recognized as the antibiotic of choice for the treatment of these infections [2], in particular when the bacteriological diagnosis has been established [3]. Meningococcal strains resistant to penicillin G remain very rare and are associated with a production of β-lactamases, while the intermediate strains are very widely described in different countries [4]. Penicillin binding protein (PBP) are conserved proteins that play an important role in the biosynthesis of the peptidoglycan of the cell wall of many pathogenic bacteria [5,6].

Analysis by SDS-PAGE (sodium dodecyl sulfate polyacrylamide gel electrophoresis) showed that *N.m* contains three defined PBP genes, including PBP 1 coding for PonA, PBP 2 coding for penA and PBP 3 coding for Pbp3 [7]. The decreased sensitivity to penicillin G has been reported as being mainly due to mutations in the structure of the PBP 2 protein encoding the penA gene [8,9]. The objective of this study is to evaluate the correlation between the expression of the altered PBP 2 gene (mosaic gene) and the decreased sensitivity to penicillin G of *N.m* strains isolated between 2010 and 2021 in hospitalized patients.

## Methods

**Study design:** this is a retrospective study conducted between 2010 and 2021 at the Microbiology laboratory of the IR-UHC in Casablanca and at the Bacteriology laboratory of the UHC Mohammed VI in Marrakech. These strains were analyzed in the laboratory of Invasive Meningococcal Infections of the Pasteur Institute of Morocco (IPM).

**Study population:** the study concerned all the services of the University Hospital Center of Casablanca and Marrakech (Morocco). Isolated samples were collected from cerebrospinal fluid (CSF) and blood cultures. Patient data consisted of age, year of isolation, ward of isolation, serogroup and MIC.

**Isolate collection and identification:** in total, 190 *N.m* isolates were collected. Twenty-three isolates with decreased sensitivity to penicillin G (0.12 ≤ MIC ≤ 0.25 µg/L) were selected for the molecular characterization of the PBP 2 gene by PCR followed by Sanger sequencing. All the strains were transplanted onto a chocolate + Polyvitex agar (bioMérieux, Marcy-l'Etoile, France). The identification was carried out according to standard bacteriological techniques.

**Penicillin G sensitivity testing:** the minimum inhibitory concentration (MIC) for penicillin G was determined for all isolates using the E-test strips (Oxoid Thermofisher United Kingdom) on Mueller-Hinton agar supplemented with 5% sheep's blood (bioMérieux, Marcy l'Etoile, France). The incubation was carried out at 37°C under a 5% CO2 atmosphere for 16 to 18h. The interpretation was made according to the recommendations of the European Committee on Antimicrobial Susceptibility Testing (EUCAST) depending on the year of isolation [10].

**DNA extraction:** DNA extraction from *N.m* strains was carried out by the QIAmp® DNA Minikit extraction kit (Qiagen, Hilden, Germany) according to the manufacturer's recommendations.

**PCR serogroup:** the serogroup of the *N.m* strains was confirmed by final time PCR [11,12].

**PCR PenA:** the reaction mixture was carried out in a final volume of 30 µl containing: MgCl 2 (5 mM); 10X PCR buffer; DNTPs (5mM); Oligonucleotides specific for penA-1F/penA-1R gene (2 mM); TaqDNA polymerase (Invitrogen Thermo Fisher Scientific) 5U/µl. The PCR search for the penA gene was carried out with the use of specific primers of a 520 bp fragment of the penA gene: penA-1F (5'GTTTTCCCAGTCACGACGTTGTAATCGAACAGGCGACGATGTC-3')/penA-1R (5'TTGTGAGCGGATAA CAATTTCGATTAAGACG GTGTTTTGTCGG-3'). All final time PCRs were performed in the Applied Biosystems Veriti 96-well thermal cycler. The PCR amplification protocol was changed as follows: Initial denaturation at 92°C for 30 seconds followed by 20 cycles of amplification of 92°C for 30 seconds and one 30 seconds at 62°C.

**PenA gene sequencing:** PCR products were purified using the Ex'S-Pure kit (Image BV, Netherlands) with two hydrolytic enzymes; Exonuclease (500 µL) and Alkaline Phosphatase (SAP 500 µL) Ex'S-Pure according to the manufacturer's instructions (one cycle of 37°C for 15 min and 90°C for 10 min). Sequencing of PCR products was carried out on the ABI 3700 DNA analyzer sequencer (Applied Biosystem) with the BigDyeF® Terminator v1.1 kit (Applied Biosystems, Foster City, USA), 10X buffer, and enzymatic purifier. 25 amplification cycles are carried out on the thermal cycler; one cycle corresponds to: A DNA denaturation step at 96°C for 10 seconds. A hybridization step at 50°C for 5 seconds. A DNA elongation step at 60°C for 4 minutes [13].

**Data collection:** the alignment of multiple sequences of the terminal part of the penA gene and the corresponding deduced amino acid sequences was carried out by the BioEdit software (version 7.2.5). The construction and visualization of the phylogenetic tree were carried out with the programs RAxML (Randomized Axelerated Maximum Likelihood) v8.2.12 and Figtree v1.4.2 respectively. The positions of the nucleotides of the penA gene and the PBP2 amino acids are compared with the reference genome of *N.m* MC58 (GenBank accession number AE002098).

**Ethical considerations:** the isolates were obtained as part of the routine activities of the microbiology laboratory of Ibn Rochd University Hospital Center (IR- UHC) in Casablanca and the Bacteriology laboratory of the UHC Mohammed VI in Marrakech. Demographic data were extracted from the computer database anonymously. Approval of ethics and informed consent were therefore not necessary. The IMD case was defined as isolation of a *N.m* or meningococcal DNA detection from a normally sterile site, such as blood, CSF or articular liquid sample.

## Results

**General characteristics of meningococcal isolates:** of the 190 *N.m* strains in our study, 66.84% of the strains were isolated from cerebrospinal fluid (CSF) and 33.15% of isolates came from blood cultures. The IMIs are mainly due to serogroup B with 93.15% (n=177) of cases, followed by serogroup W with 3.70%. Only 1.57% of the isolates are from serogroup Y and 1.57% are from serogroup C. Of the strains analyzed, 143 isolates (75.26%) are sensitive to penicillin G (MIC ≤ 0.06 µg ml -1) and 24.73% (n = 47) have a reduced sensitivity (0.12 ≤ MIC ≤ 0.25 µg ml -1), most of them were from serogroup B (n=40). Of the 47 strains that phenotypically show reduced susceptibility to penicillin G, 23 (49%), show the penA gene with all five mutations, while 24 strains (51%) show no pbp2 mutation on the penA gene.

**Characteristics of the penA:** the amino acid sequences deduced from PBP2 (amino acids 298 to 581) were aligned in comparison with the reference strain MC58. Five mutations in 23 strains were observed at the level of pbp2 (Table 1). These modified positions were located around the preserved KTG pattern.

**Table 1:** molecular characteristics of the 23 *Neisseria meningitidis* strains sequenced by Sanger sequencing

Number of isolates	Serogroup	City	Isolation service	Age	Year of isolation	MIC
1	B	Casablanca	PMW	8 M	2019	0.25
2	B	Casablanca	EICU	12 M	2017	0.25
3	B	Casablanca	EPW	8 M	2013	0.25
4	B	Casablanca	EPW	13 M	2018	0.25
5	B	Casablanca	PMW	7 M	2015	0.25
6	B	Casablanca	PMW	8 M	2016	0.25
7	B	Casablanca	EICU	23 Y	2018	0.25
8	B	Casablanca	EICU	4 Y	2012	0.125
9	B	Casablanca	EICU	17 Y	2014	0.125
10	B	Casablanca	PMW	5 Y	2015	0.12
11	B	Casablanca	PMW	3 Y	2015	0.25
12	B	Casablanca	PMW	5 Y	2015	0.25
13	B	Casablanca	PMW	1 Y	2015	0.25
14	B	Casablanca	EICU	40 Y	2015	0.25
15	B	Casablanca	PMW	2 Y	2015	0.25
16	B	Casablanca	PMW	2 Y	2015	0.25
17	B	Casablanca	PMW	3 Y	2016	0.25
18	B	Casablanca	EPW	5 Y	2016	0.25
19	C	Casablanca	EICU	26 Y	2011	0.125
20	W	Casablanca	EICU	8 Y	2016	0.25
21	B	Marrakech	PMW	13 Y	2017	0.12
22	B	Marrakech	PMW	2 Y	2014	0.25
23	Y	Marrakech	PMW	7 Y	2016	0.25

PMW: Pediatric Medical Ward; EICU: Pediatric Intensive Care Unit; EPW: Emergency Pediatric Ward; MIC: Minimum Inhibitory Concentration; M: Month; Y: Year.

**Figure 1:**
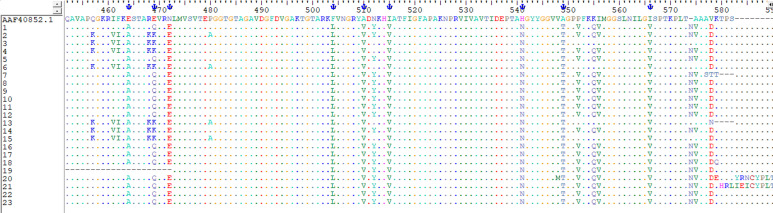
the multiple alignment of the amino acid sequences in strains with reduced sensitivity to penicillin G with that of the penA gene of the wild-type strain

**Figure 2:**
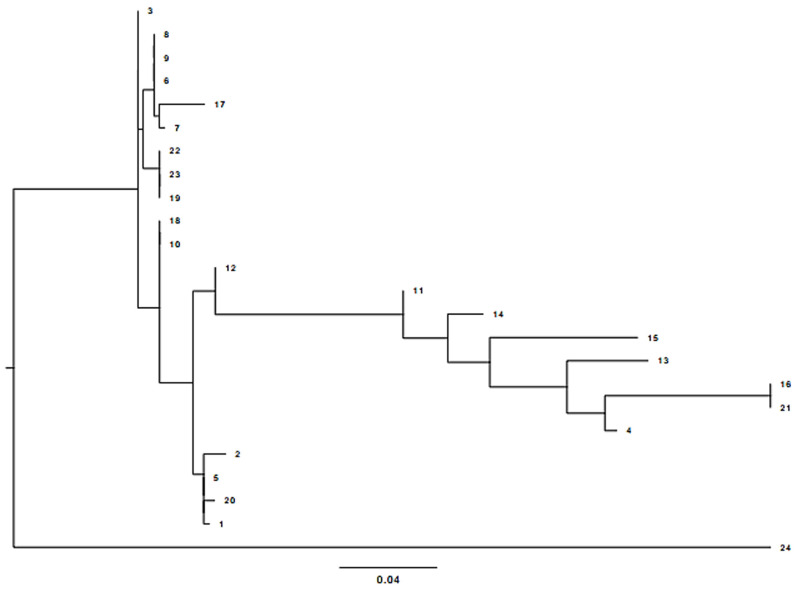
schematic illustration of the phylogenetic analysis of the 23 *Neisseria meningitidis* strains isolated compared to the wild strain

In this work, all Pen_I isolates studied, the change from amino acid Ile 566 to Val 566 (ATT to GTT) was detected at the 3' end of the PenA gene. There are four additional nucleotide transitions involving amino acid substitution, such as Phe 504 to Leu, Ala 510 to Val, Ile 515 to Val and His 541 to Asn (Figure 1). Of the set of isolates decreased susceptibility to penicillin G, 49% (n=23) of the strains analyzed had penA mosaic alleles and five amino acid substitutions (F504 L, A510 V, I515 V, G541 N and I566 V) were found in the transpeptidase region of PBP2 encoding the penA gene. Thus, 51% of the isolates sensitive to penicillin G did not carry the mosaic gene and did not contain amino acid substitutions in the PBP2 protein. The absence of the penA mosaic gene coding for the five amino acid changes associated with reduced sensitivity to penicillin G, does not necessarily indicate a penicillin G sensitive phenotype. The phylogenetic tree shows that the sequences of the penA gene are genetically diversified, and composed of four subgroups distant from the wild-type MC58 strain (Figure 2).

## Discussion

Invasive meningococcal disease is a worldwide public health problem, representing a major cause of morbidity and mortality. In our results, we noted that the MICs of strains with mutations coding for the five modified positions in PBP2 are increased (0.25 mg/l < MIC < 0.5 mg/l), while those strains without penA gene mutations have lower MICs.

In the mid-1980s, *N.m*'s resistance to antibacterial agents showed an important inflection point strains with reduced sensitivity to penicillin, which was caused by alterations in penicillin-binding protein (PBP) [14]. We know that penicillin has long been the antibiotic of choice for the treatment of meningococcal infections. The antibiotics of the β-lactam family are the most widely used, due to their good activity and with very limited side effects. In *N.m*, there are two mechanisms of resistance to penicillin; the first is linked to resistance by the production of β-lactamase, this mechanism characterizes some strains with an MIC greater than 1 mg/l [15]. The second resistance mechanism present in strains with a decreased sensitivity to penicillin is related to the decrease in the affinity of penicillin-binding proteins (pbp). The analysis of the sequences of the genes coding for the PBPs shows the presence of mosaic genes [15].

In the present work, 24.7% (n = 47) of the isolates are of reduced sensitivity (0.12 ≤ MIC ≤ 0.25 μg ml -1), this rate of decreased susceptibility strains is comparable to that of Portugal 24.6% [16]. The proportions of isolates exhibiting reduced sensitivity to *N.m* penicillin G are higher in France at 31.7% [17] and higher at 55.3% in a Spanish study [18]. In Italy, before 2002, Pen I meningococci represented 7.5% of isolates, but since then the percentage has increased to 27.4% in 2003 [19]. The strains with a decreased sensitivity to penicillin G (n=23) had penA mosaic alleles and had five amino acid substitutions (F504 L, A510 V, I515 V, G541 N and I566 V). Similar results were found in Tunisia where the majority of isolates had a reduced sensitivity to penicillin G, with a correlation between the values of resistance to penicillin and the modified penA alleles [20].

In the present work, among the 47 strains that phenotypically present a reduced sensitivity to penicillin G, 23 (49%) present the penA gene with the five mutations. Similar results were found in a large European study where the authors collected approximately 1600 isolates collected mostly from Europe, 65% showed reduced susceptibility to penicillin G, but only 38% had mosaic penA alleles [21]. In another study, on isolates of *N.m* with reduced penicillin susceptibility, du Plessis and his collaborators identified the mosaic penA gene in only 25 of 87 (28.7%) isolates with intermediate penicillin susceptibility [22]. In the USA, of the 466 *N.m* isolates collected, 10.3% (n=48) strains had reduced susceptibility to penicillin G, among of these, 63% had penA mosaic alleles, which contained all five amino acid changes [23]. In a German study, seven of 22 *N. meningitidis* G isolates and 19 of 75 N. lactamica strains with intermediate sensitivity to penicillin had five specific amino acid mutations in the transpeptidase domain of PBP2. The majority of intermediate strains (n = 53) of N. lactamica had only three amino acid alterations (I515 V, G541 N, and I566 V) instead of five in PBP2 [24].

In the United States between 2012 and 2016, of the 695 strains collected and characterized by whole genome sequencing, 208 isolates were of intermediate susceptibility to penicillin G, 78.8% (n=164) showed the presence of at least 4 mutations characterized in mosaic penA alleles (F504L, A510V, I515V, H541N or I566V) [25]. Reduced susceptibility to penicillin G may result from other, as yet unidentified mechanisms in addition to mutations in the penA gene. Reduced PBP-1 affinity for penicillin G and decreased expression of class 3 porin have been shown to increase penicillin G resistance in *N.m* [26].

The limitation of this study is related to the small sample size. The present work concerns only 23 strains with reduced sensitivity to penicillin G. The aim of this study was to evaluate the relationship between the reduced sensitivity to penicillin G of *N.m* strains and the expression of the altered PBP 2 gene. We suggest that future studies be conducted to evaluate other mechanisms that may reflect reduced sensitivity to penicillin G such as whole genome sequencing of *N.m*.

## Conclusion

Invasive meningococcal infections are mandatory to report in Morocco; these infections require immediate diagnosis and appropriate prophylaxis. Correlation between the penA gene sequence and penicillin MIC identified mosaic structures clearly associated with reduced sensitivity. However, this is not the only mechanism that can reflect this reduced sensitivity to penicillin G but there are other mechanisms that have been described, hence the need to increasingly refer to whole genome sequencing (WGS) of *N.m* to understand this entire mechanism.

### What is known about this topic

- *Meningococcal meningitis is a serious public health problem;*
- *The mechanisms of penicillin resistance in N.m is linked to both resistance through β-lactamase production and decreased affinity of penicillin-binding proteins (pbp);*
- *Decreased susceptibility to penicillin G has been reported to be due to mutations in the structure of the pbp 2 protein encoding the penA*.

### What this study adds

- *The frequency of N.m strains with reduced sensitivity to penicillin G has increased in recent years;*
- *Monitoring the relationship between penicillin MICs and penicillin sequence analysis is crucial for more effective antimicrobial and prophylactic treatment of patients and their contacts*.
